# Novel Cavity Disinfectants Containing Quaternary Ammonium Monomer Dimethylaminododecyl Methacrylate

**DOI:** 10.3390/ma9080674

**Published:** 2016-08-09

**Authors:** Wen Zhou, Biao Ren, Xuedong Zhou, Hockin H.K. Xu, Michael D. Weir, Mingyun Li, Mingye Feng, Jiyao Li, Xin Xu, Lei Cheng

**Affiliations:** 1 State Key Laboratory of Oral Diseases, Sichuan University, Chengdu 610000, China; zhouwendentist@139.com (W.Z.); renbiao@scu.edu.cn (B.R.); zhouxd@scu.edu.cn (X.Z.); limingyun@scu.edu.cn (M.L.); fengmy@stanford.edu (M.F.); jiyao_li@aliyun.com (J.L.); 2 Department of Operative Dentistry and Endodontics, West China Hospital of Stomatology, Sichuan University, Chengdu 610000, China; 3 Biomaterials & Tissue Engineering Division, Department of Endodontics, Prosthodontics and Operative Dentistry, University of Maryland Dental School, Baltimore, MD 21201, USA; Hxu@umaryland.edu (H.H.K.X.); MWeir@umaryland.edu (M.D.W.)

**Keywords:** dimethyl aminododecyl methacrylate, chlorhexidine digluconate, dentin bond durability, MMPs inhibition, antibacterial, saliva microbial-aging

## Abstract

This study was set to assess the possible benefits of novel cavity disinfectants with 5% dimethylaminododecyl methacrylate (DMADDM); and compare the effectiveness of saliva microbial-aging method with water-aging in measuring the changing of resin–dentin bond strength. Three cavity disinfectants were tested: 0.2% Chlorhexidine (CHX); 5% DMADDM; and 5% DMADDM + 0.2% CHX. Microtensile bond strength (μTBS) test was performed after microbial-aging with saliva microbial or water aging for one month. Hydroxyproline (HYP), the production of collagen degradation, was measured spectrophotometrically. Additionally, the antibacterial effects of each reagent were evaluated. The 5% DMADDM exerted the least percentage of resin–dentin bond strength loss after one month microbial-aging (*p* < 0.05). There were no statistically significant differences of bond strength decrease after one month water aging among the tested groups (*p* > 0.05). Microbial-aging method yield more drop of bond strength than water aging in all groups except 5% DMADDM (*p* < 0.05). Meanwhile, 5% DMADDM had the same matrix metalloproteinases (MMPs) inhibitory effects as the other two agents (*p* > 0.05), but much stronger antibacterial capability than 0.2% CHX (*p* < 0.05). This indicated that a cavity disinfectant with 5% DMADDM is promising for improving the stability of resin–dentin bonds in appearance of saliva biofilm; and the saliva microbial-aging method is more promising for studying the durability of resin–dentin bonds than water aging.

## 1. Introduction

Resin composites are widely used as filling materials [1]. However, the failure rate of resinous restorations is relatively high [2]. Bacterial activity has proven to be the main causative factor for placement and replacement of restorations [3]. Modern minimally invasive techniques leaving behind the affected-dentin so that the cavities likely containing more residual microorganisms [4]. The residual bacteria in the tooth cavity can magnify the problems associated with microleakage and lead to pulp inflammation [5,6]. Moreover, bacteria will invade along the tooth-restoration margins due to microleakage [7,8], resulting in secondary caries [2,4,9].

The resin–dentin bond strength will deteriorate over time due to the hydrolysis of collagen matrix of the hybrid layers [10,11]. Demineralized dentin collagen matrix and infiltrated resin form the hybrid layer, which is paramount to dentin bonding strength [12]. Proteolytic degradation of hybrid layer by host-derived matrix metalloproteinases (MMPs) is believed to be among the major reasons for the failure of resin restorations [9,13]. Weak acids such as lactic acid and enzymes produced by cariogenic bacteria can activate MMPs, impairing resin–dentin bond durability [14,15].

Cavity disinfectants are used to deal with above problems. The ideal dentin disinfectant should combine the possession of a potent antimicrobial action and inhibitory effect of the MMPs.

It has been demonstrated that Chlorhexidine (CHX) have broad spectrum antibacterial activity and can inhibit the catalytic activity of MMPs, so CHX is recommended used as cavity disinfectant to improve resin–dentin bond durability [16,17]. However, CHX could not completely eliminate the viable bacteria in the cavity. As CHX is released, its antibacterial and MMPs inhibition effects may be weakened [4,18].

Dimethylaminododecyl methacrylate (DMADDM) is a new quaternary ammonium methacrylates (QAMs) monomer. It has excellent antibacterial and MMPs-inhibitory effects [4]. Previous studies showed that when DMADDM is incorporated into adhesive systems, it can exert antibacterial and MMPs inhibition effects to the parent solution, without compromising the dentin bond strength [19,20,21,22]. It can be immobilized by polymerization in bonding agents, which would not leach out from the hybrid layer. Thus, the modified adhesives could act as a primer, cavity disinfectant and long-term antibacterial and MMPs-inhibitory agent [23,24]. Previous research studied DMADDM by adding it into commercially available products such as adhesive systems, resin composites, glass–ionomer cements and so on, modifying the wanted properties of these products [4,25,26]. However, it is relatively complex to prepare such experimental materials, and these modified materials have not involved in pretreatment of the dentin before the adhesive process. Moreover, there is no study on the effectiveness of DMADDM used independently as cavity disinfectant. As mentioned above, the widely used cavity disinfectant CHX has some drawbacks, including that it cannot successfully manage through the problems of resinous restorations encountered. Thus, this study was set to design a novel cavity disinfectant containing DMADDM and to find its possible benefits to the dentin bond durability.

Water aging is one of the most popular ways to study resin–dentin bond strength. This method has not taken the influence of bacteria into consideration. To deal with this drawback, the authors applied a saliva microbial-aging method: the microtensile specimen was covered by saliva biofilm, incubating in a modified artificial saliva medium with cysteine (Mcbain medium).

Accordingly, the objectives of this study were to investigate the possibility of using novel cavity disinfectant containing DMADDM to improve the stability of resin–dentin bond strength, as well as compare the effectiveness of saliva microbial-aging method with water aging in measuring the changing of resin–dentin bond strength for the first time.

## 2. Results

**Figure 1:**
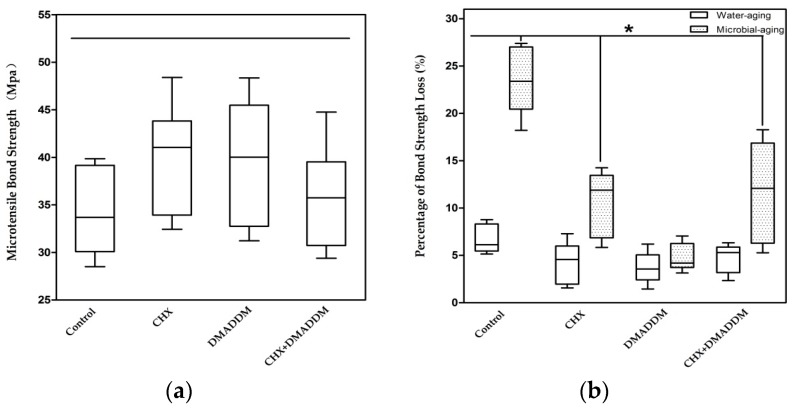
Microtensile Bond testing: (**a**) The microtensile bond strength without aging treatment; and (**b**) the percentage of microtensile bond strength lost after water aging and microbial aging (*n* = 6). The asterisk represents statistically significant differences; * *p* < 0.05.

Figure 1a plots the microtensile bond strength without aging treatment. There were no statistically significant differences among the four groups. Figure 1b shows the percentage of decreased microtensile bond strength after water aging and saliva microbial aging (*n* = 6). The percentage of bond strength loss due to microbial aging was much higher than water aging in all groups except DMADDM (*p* < 0.05). Moreover, after one-month water aging, there were no differences among the four groups (*p* > 0.05), for microbial aging, the percentage of decreased bond strength in DMADDM group was the lowest (*p* < 0.05).

**Figure 2:**
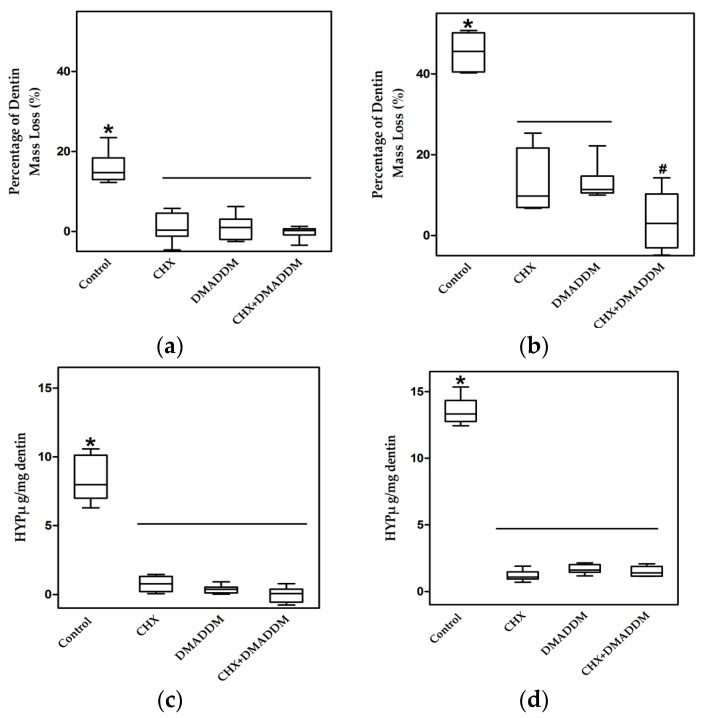
Dentin beams proteolytic degradation assay. The percentage of dry mass loss after: (**a**) 7 d (*n* = 6); and (**b**) 30 d. The amount of dissolved collagen from demineralized dentin beams after: (**c**) 7 d; and (**d**) 30 d (*n* = 6). The Y-axis represents the measured mass (μg) of dissolved hydroxyproline at 7 or 30 d/dentin dry mass (mg) at 0 d. The asterisk represents statistically significant differences; * *p* < 0.05.

The percentage of dentin mass loss at seven and 30 days is shown in Figure 2a,b, respectively (*n* = 6). Dentin incubated in complete storage medium (CM) without any inhibitor lost 18.16% (7 d) and 45.46% (30 d) of the dry mass. Dentin mass losses of the last three groups were significantly less than control group (*p* < 0.05) and not significantly different to each other (*p* > 0.05), yielding a loss of 1.00% (7 d) and 13.19% (30 d) for CHX group, 1.00% (7 d) and 12.99% (30 d) for DMADDM group, and 0.25% (7 d) and 3.67% (30 d) for DMADDM + CHX group.

The dissolution of collagen peptides determined by hydroxyproline (HYP) analysis is shown in Figure 2c,d (7 d and 30 d, respectively) (*n* = 6). Hydroxyproline dissolution weights (μg) per 1 mg dry weight of collagen matrix of the three groups were significantly lower than the control group (*p* < 0.05): 0.8 (7 d) and 1.18 (30 d) for CHX group, 0.30 (7 d) and 1.64 (30 d) for DMADDM group, 0.20 (7 d) and 1.45 (30 d) for DMADDM + CHX.

**Figure 3:**
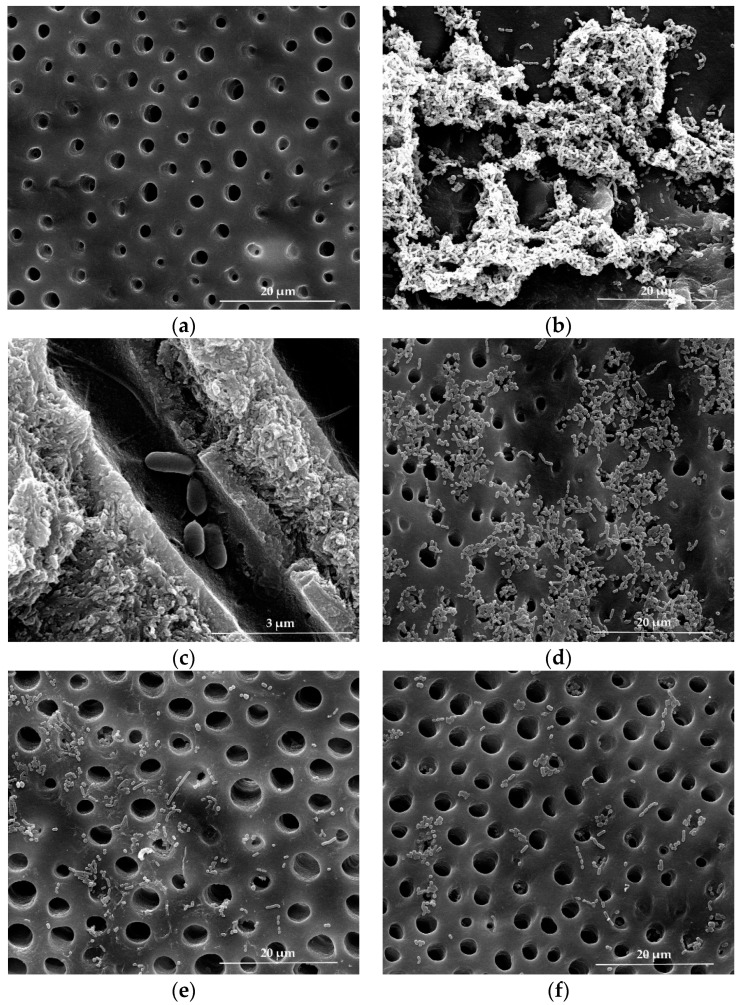
Scanning electron microscopy (SEM) micrographs of bactericidal effect on dentin caries related bacteria. (**a**) Dentin surface before bacteria impregnation; (**b**) bacteria impregnation on the surface of the dentin; (**c**) bacteria impregnation into dentin tubules; (**d**) the bacteria treated with 0.2% CHX; (**e**) 5% DMADDM; and (**f**) 5% DMADDM + 0.2% CHX.

Figure 3 shows typical scanning electron microscopy (SEM) micrographs of different agents on dentin caries related bacteria. Figure 3a shows the dentin blocks before bacteria impregnation. Dentin caries related bacteria impregnated in dentin are shown in Figure 3b. In Figure 3c, bacteria in dentinal tubules on the cross-section of dentin are shown when the dentin was opened after bacteria impregnation, showing successful bacteria impregnation into the interior of dentin. In Figure 3d, dentin blocks treated with CHX had much more remaining bacteria. Dentin with DMADDM and DMDADDM + CHX showed the least bacteria.

**Figure 4:**
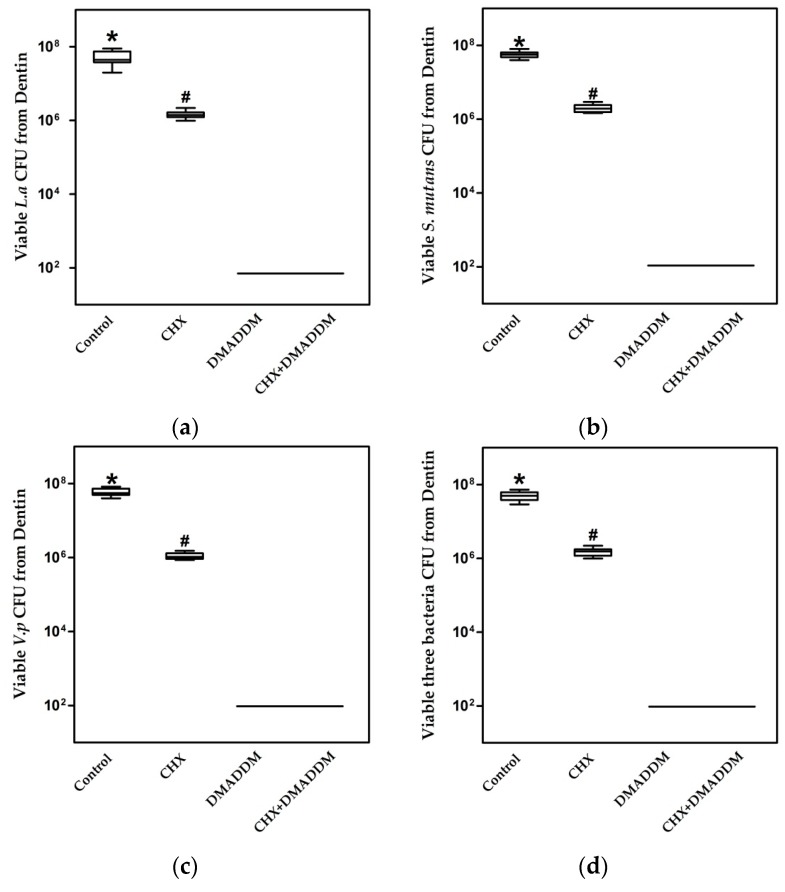
Colony-forming units (CFU ) of bacteria in dentin blocks harvested by sonication (*n* = 10): (**a**) *Lactobacillus acidophilus*; (**b**) *S. mutans*; (**c**) *Veillonella parvula*; (**d**) Mixture of three bacteria. The asterisk represents statistically significant differences; * *p* < 0.05.

These qualitative observations were corroborated with quantification of bacteria colony-forming units (CFU) in dentin blocks in Figure 4 (*n* = 10). The CFU was the highest in the control dentin blocks without treatment. DMADDM and DMADDM + CHX eliminate almost all of the bacteria in dentin blocks. The group of CHX showed the least bactericidal activity, reducing the CFU by no more than two orders of magnitude.

## 3. Discussion

To improve the durability of resinous restorations, cavity disinfectants should possess antimicrobial action, MMPs inhibitory effect and durability.

CHX is one of the gold standard antimicrobial agents and matrix metalloproteinase inhibitors [27]. CHX has a broad antibacterial spectrum. It is effective against Gram-positive microbes but weaker against some kind of Gram-negative ones [10,28]. Therefore, CHX application before placement of the restoration has been recommended to reduce the residual microflora in the prepared tooth cavity [10].

Demineralized dentin collagen matrix acts as a scaffold for resin infiltration during the resin–dentin bonds procedure [12]. Degradation of collagen matrices by MMPs is one of the major reasons for the failure of resin restorations [29,30]. Acid-etchants used in dentin bonding and weak acids released by cariogenic bacteria can uncover and activate matrix-bound MMPs. Incomplete resin infiltration also contributes to their activation [10,31]. The mechanism of CHX on MMPs inhibition is probably based upon the cationic–anionic reaction of CHX, which may deform negatively charged MMPs molecules and prevent them from binding to substrates [32]. Moreover, the cation chelation effect of CHX binding with calcium and zinc ions of MMPs is another inhibition mechanism [33].

However, CHX pre-treatment can be problematic, even though 0.002% CHX is sufficient to prevent host-derived MMPs from degrading exposed collagen fibrils [28], and its cationic properties enable it to bind to phosphorate groups in apatite, producing a strong affinity for tooth surfaces [10,34]. Part of the CHX may be washed out from primed dentin during application of liquid comonomers for bonding and, because of CHX is water soluble [35,36], as it releases, it may lose its antibacterial and MMP inhibitory abilities. The effective concentrations of CHX have varied between 0.002% and 4%, 0.2% and 2% being the most common concentrations used [9]. However, some studies have suggested that relative high concentration of (2%) CHX digluconate solution adversely affects the bond strength of self-etching and self-adhesive adhesive systems [10,37]. Thus, the concentration of 0.2% was chosen in this study. The minimal bactericidal concentration (MBC) and Minimum inhibitory concentration (MIC) of CHX is 6 μg/mL and 2 μg/mL, respectively, which is similar to DMADDM, 12 μg/mL and 6 μg/mL [38], respectively, but 0.2% CHX is much lower than the 5% DMADDM concentration. As is shown in quantification of bacteria CFU in dentin blocks, 10 μL disinfectants on the dentin blocks were diluted into 2 mL cysteine peptone water (CPW) after disinfection process. Thus, the concentration of CHX was 0.01 μg/mL and DMADDM was 0.25 μg/mL, far from the MIC and MBC of each agents. The following serial dilution decreased the concentration further. Thereafter, the residues of the disinfectant were not able to kill or inhibited bacteria. As the disinfectants were not inactivated, it was unsure whether the bacteria were killed or inhibited. However, the results still indicated that the bactericidal effectiveness of 0.2% CHX was the lowest when compared to 5% DMADDM and the combination of these two agents. In addition, the CHX solutions are highly cytotoxic to cultured cells, even at the concentration of 0.01% [39].

DMADDM is a kind of quaternary ammonium methacrylates. It has potent antimicrobial activity for its positive charge, thus can change the membrane permeability or surface electrostatic balance of the negatively charged bacteria, leading to cytoplasmic leakage [4,40]. Besides the ability to kill bacteria, the cationic DMADDM can electrostatitically block the negatively charged catalytic sites of MMPs [41]. Many previous studies demonstrated that the addition of 5% DMADDM into adhesive systems had no adverse effect on adhesive bonding strength [42,43]. Additionally, DMADDM could be immobilized by polymerization in bonding agents and would not leach from the hybrid layer [15]. Moreover, DMADDM had much lower cytotoxicity, even lower than BisGMA [41]. DMADDM was used to modify dental materials in many studies. However, it is relatively complex to prepare such experimental materials. These modified materials have not been involved in pretreatment of the dentin before the adhesive process. Moreover, there is no study on the effectiveness of DMADDM used as cavity disinfectant. Thus, in the current study, DMADDM was incorporated with deionized water or 0.2% CHX as a new cavity disinfectant to deal with the shortcomings of the existing disinfectants.

CHX and DMADDM were physically mixed in the cavity disinfectant. However, the combination showed no extra benefit when compared with DMADDM alone. DMADDM is in the state of monomers before polymerization. In a study by Hiraishi et al., it is speculated that CHX could interfere with the function of adhesive monomers [10]. Therefore, CHX might adversely affect the function of DMADDM. In addition, CHX has strong affinity for tooth surfaces [10,34]. When the dentin surface was treated with the mixture of CHX and DMADDM, the binding with DMADDM and MMPs in the hybrid layer were more or less hampered.

Assessment of hydroxyproline, collagen fragments that are produced through the action of MMPs on type I collagen, is a quantitative way to measure MMP-inhibitory ability of materials [44]. Loss of dry mass over time provides an indirect measurement of solubilization of matrix by endogenous MMP activity [45]. The results of this study showed that 5% DMADDM has the same MMPs inhibitory capacity as 0.2% CHX and their combination. As DMADDM can polymerize in bonding systems, it might be capable of providing long term MMPs inhibitory effects that will not decrease with time.

In comparison, DMADDM achieved antibacterial effects much stronger than CHX alone, and the same as the combination of DMADDM and CHX. All four CFU counts in dentin disk with DMADDM or dual agents were six orders of magnitude lower than that of CHX alone. The results of SEM also showed the same trend. Therefore, it would be more beneficial to use DMADDM alone or combine DMADDM with CHX to obtain a greatly increased antibacterial potency compared to CHX.

Microtensile bond strength (μTBS) methodology was introduced in 1994, and is widely used to measure bond strength [46,47]. Via reducing specimen size, this test successfully reduced the number and size of flaws, better reflecting the actual interfacial bond strength to dentin [48]. When it comes to investigating the durability of resin–dentin bonds, the μTBS specimens incubated in water often presented without the appearance of bacteria [21]. Once the resin restorations have been placed in tooth cavities, the restorations were in the oral ecosystem. Biofilm will form on the restoration surfaces, and bacteria will invade along the tooth-restoration margins due to microleakage. Weak acids such as lactic acid and enzymes produced by cariogenic bacteria can activate MMPs, impairing resin–dentin bonds durability [14,15].

It is well known that in vitro artificial aging techniques are carried out to simulate in service conditions [49]. They are proposed to accelerate the degradation of the resin–dentin interface, and hence enable the measurement of the long term bonding and durability of dental materials [50,51]. In this study, saliva microbial-aging method was used to measure the changing of resin–dentin bond strength with appearance of bacteria. The μTBS specimens were incubated in McBain medium with saliva biofilm. The results revealed that resin–dentin bond strength fell more significantly in one month of microbial-aging storage than water aging. After one-month water aging, the amount of decreased bond strength of all groups was limited and showed no statistical difference. Generally, it takes at least six months for water aging to cause an obvious drop of resin–dentin bond strength [21,52]. By contrast, with the process of one-month microbial aging, all groups except DMADDM exhibited more decreased bond strength and statistically significant differences with the water aging specimens. The reasons of bond strength deterioration during water aging mainly involve in the swelling and plasticizing of the adhesive resin, as well as degradation of collagen matrices [52]. As for microbial aging, apart from the reasons above, bacteria induced changes such as fluctuation of pH and the production of bacterial metabolite also contribute to the decreased bond strength. It seems that microbial-aging method successfully accelerated the deterioration of bond strength, thus more effectively detecting the difference in stability of bond strength between groups. This method seems promising for studying the durability of resin–dentin bonds.

However, the present study was performed in a static environment. Clinically, numerous other factors affect bond strength such as thermal, mechanical, chemical and fatigue stresses during function. Thus, further in vitro studies that simulate the real clinical environment are needed.

## 4. Materials and Methods

The extracted human caries-free third molars used in this study were obtained with patient’s informed consent under a protocol approved by the West China Hospital of Stomatology, Sichuan University (ethics committee approval number: VWCHSIRB-D-2015-131). These teeth were stored in 0.01% thymol solution for no more than 1 month before use. The following experiments were performed at least three times.

### 4.1. Cavity Disinfectants Preparation

DMADDM was synthesized as described in previous studies [21,38], and incorporated into deionized water or 0.2% CHX at a mass fraction of 5% by agitation. Moreover, Fourier transform infrared spectroscopy (Nicolet 6700, Thermo Scientific, Waltham, MA, USA) was used to verify the physical mixture of CHX and DMADDM.

### 4.2. Microtensile Bond Testing

The enamel and superficial dentin of the third molars were removed from the crown with a diamond saw to prepare a flat mid-coronal dentin surface. The surfaces were further polished with a diamond bur for 60 s in order to standardize the smear layer [53,54].

The teeth were distributed into four groups according to the cavity disinfectants used: (1) no disinfection treatment; (2) 0.2% CHX, a cotton pellet saturated with 0.2% CHX, with a dwell time of 20 s, after that the dentin surface was blown dry; (3) 5% DMADDM; and (4) 5% DMADDM + 0.2% CHX. For the last two groups, the dentin surface was disinfected with respective solution in the same way as Group 2. After pre-treatment of the dentin, the primer of SE Bond (Kuraray Medical, Tokyo, Japan) was applied with a brush-tipped applicator and rubbed for 20 s and dried with a stream of air for 5 s. Adhesive was spread on the dentin surface and light-cured for 10 s. Then, composite blocks were prepared on the teeth surface. The bonded specimens were stored in water at 37 °C for 24 h. Thereafter, the bonded teeth were sectioned occlusogingivally into 1 × 1 × 10 mm^3^ composite dentin sticks.

Seventy-two resin–dentin sticks in each group were treated in three different ways (*n* = 6): (1) subjected to microtensile test immediately; (2) stored in water at 37 °C for 1 month; and (3) stored in McBain broth with saliva biofilm at 37 °C for 1 month, changing the incubating media once a day. Saliva for incubation was obtained from seven healthy female and three male volunteers, ranging in age from 23 to 25. The volunteers had no history of extant periodontal disease and not taken antibiotics for the previous 3 months [55].

At the specified time point, individual sticks were fixed on a testing Machine, and then subjected to crosshead tensile forces at a speed of 0.5 mm/min until failure [40,56].

### 4.3. Dentin Beams Proteolytic Degradation Assay

The enamel and superficial dentin of the molars were removed from the crown with a diamond saw. One-millimeter-thick dentin disks of mid-coronal dentin were then prepared. Dentin beams 6 × 2 × 1 mm^3^ were sectioned from each dentin disk.

The dentin beams were submerged in 10% phosphoric acid for 18 h at 25 °C to demineralize them. In order to remove phosphoric acid, the dentin beams were incubated in deionized water for 72 h at 4 °C under constant stirring, and the water was changed every 16 h. Then, they were dried over anhydrous calcium sulfate for 8 h, and the mass of each dentin beam was measured by a digital microbalance. The beams were rehydrated for 24 h in 0.9% NaCl containing 10 U·mL^−1^ of penicillin G and 300 μg·mL^−1^ of streptomycin (pH 7.0). Radiography was used to ensure the absence of residual minerals [13].

Forty-eight dentin beams were distributed into four groups and placed in Eppendorf tube, incubated with 500 μL different types of media at 37 °C for 7 or 30 days (*n* = 10): (1) a calcium- and zinc-containing complete storage medium CM, containing 5 mM HEPES, 2.5 mM CaCl_2_·H_2_O, 0.05 mM ZnCl_2_, and 0.3 mM NaN_3_ (pH 7.4); (2) 0.2% CHX in CM (pH 7.4); (3) 5% DMADDM in CM (pH 7.4); and (4) 0.2% CHX + 5% DMADDM in CM (pH 7.4) [41]. After that, the media were agitated, and the 50 μL supernatants were used to determine the concentration of hydroxyproline, by spectrophotometrical measurement at 558 nm. The mass of each dentin beam was measured again, and the percentage of dry mass loss was calculated, loss of dentin mass = (dry mass at 0 d − dry mass at 7 or 30 d)/dry mass at 0 d.

### 4.4. Inhibition Effects on Bacteria Relating to Dentin Caries Impregnated in Dentin

We use the method described in Cheng’s study to judge the effects of the experimental agents on bacteria impregnated in human dentin blocks [4]. The use of *Streptococus mutans* (ATCC 700610, UA159, American Type Culture, Manassas, VA, USA), *Veillonella parvula* (ATCC 10790, American Type Culture, Manassas, VA, USA), and *Lactobacillus acidophilus* (ATCC 4356, American Type Culture, Manassas, VA, USA) [37], which are related to dentin caries, was approved by the State Key Laboratory of Oral Diseases, Sichuan University. Sixty dentin blocks of approximately 4 × 4 × 0.8 mm^3^ were cut from the crown of the extracted third molar, using silicon carbide abrasive. The dentin blocks were treated with 37% phosphoric acid for 3 min, and then rinsed with distilled water for 3 min. They were sterilized with ethylene oxide for 12 h and de-gassed for 7 days to remove the ethylene oxide gas.

Twenty-four-hour cultures of *Streptococus mutans*, *Veillonella parvula*, and *Lactobacillus acidophilus* suspension in BHI were prepared to 10^10^ CFU/mL. A 2 μL aliquot of the above bacteria suspension was placed on the surface of the dentin blocks, and the suspension was left to infiltrate into the dentin for 10 min to simulate bacterial colonization in the dentin. After impregnation of bacteria, 10 μL disinfection agents were daubed on the dentin surface according to the group criteria, for 20 s. These dentin blocks were divided into 2 groups: (1) Immersed treated and untreated blocks in 1% glutaraldehyde in PBS for 4 h at 4 °C, and then dehydrated with graded ethanol, and rinsed them with 100% hexamethyldisilazane. Sputter-coating gold was performed. Next, examined using SEM; (2) Place into 2 mL CPW in a tube, with ten blocks for each group. In order to collect the bacteria, sonication was used at a frequency of 40 kHz for 5 min, and the bacteria suspension in CPW was vortexed at the maximum speed for 20 s by a vortex mixer. The collected bacteria suspension was serially diluted in CPW, and plated on blood agar (BA) plate and two selective media: BA plate was used for measuring the whole number of the harvested live bacteria, mitis sucrose bacitracin agar (MSB) was for evaluating the number of *Streptococus mutans*, and *Lactobacillus* selective agar (LBS) for *Lactobacillus acidophilus*. The plates were incubated at 5% CO_2_ and 37 °C for 3 days, and the number of colonies was counted. The numbers of *Streptococus mutans* and *Lactobacillus acidophilus* were directly calculated by the counted CFU number, while the quantity of *Veillonella parvulathe* was determined by subtracting the number of *Streptococus mutans* and *Lactobacillus acidophilus* from total number of bacteria.

### 4.5. Statistical Analyses

Kruskal–Wallis test, followed by Dunn’s multiple comparison was performed to detect the significant effects of the variables. Statistical significance was preset at *p* = 0.05.

## 5. Conclusions

The present study investigated the effect of novel cavity disinfectants with 5% DMADDM, and compared the difference between microbial-aging method with water-aging method in measuring the stability of resin–dentin bond strength for the first time. This study demonstrated that: (1) the cavity disinfectant with 5% DMADDM might be able to improve the bond strength durability; (2) 5% DMADDM had the same MMPs inhibitory capability as 0.2% CHX and 0.2% CHX + 5% DMADDM; (3) 5% DMADDM alone or in conjunction with 0.2% CHX had antibacterial effects much stronger than 0.2% CHX alone; and (4) when compared with water-aging method, microbial-aging method was more effective for detecting the difference of stability of bond strength between groups, which makes it a promising method for studying the durability of resin–dentin bonds.
